# Correlation analysis of physical fitness and retinal microvasculature by OCT angiography in healthy adults

**DOI:** 10.1371/journal.pone.0225769

**Published:** 2019-12-03

**Authors:** Pieter Nelis, Boris Schmitz, Andreas Klose, Florian Rolfes, Maged Alnawaiseh, Michael Krüger, Nicole Eter, Stefan-Martin Brand, Florian Alten

**Affiliations:** 1 Department of Ophthalmology, University of Muenster Medical Center, Muenster, Germany; 2 Institute of Sports Medicine, Molecular Genetics of Cardiovascular Disease, University Hospital Muenster, Muenster, Germany; 3 Department of Physical Education and Sports History, University of Muenster, Muenster, Germany; Nicolaus Copernicus University, POLAND

## Abstract

Optical coherence tomography angiography (OCT-A) represents the most recent modality in retinal imaging for non-invasive and depth-selective visualization of blood flow in retinal vessels. With regard to quantitative OCTA measurements for early detection of subclinical alterations, it is of great interest, which intra- and extra-ocular factors affect the results of OCTA measurements. Here, we performed OCTA imaging of the central retina in 65 eyes of 65 young healthy female and male participants and evaluated individual physical fitness levels by standard lactate diagnostic using an incremental maximal performance running test. The main finding was that OCTA measurements of the foveal avascular zone (FAZ) area were associated with physical fitness. Using multivariate regression analysis, we found that running speed at the individual lactate threshold, a marker strongly associated with aerobic performance capacity, significantly contributed to differences in FAZ area (β = 0.111, p = 0.032). The data indicates that smaller FAZ areas are likely observed in individuals with higher aerobic exercise capacity. Our findings are also of interest with respect to the potential use of retinal OCTA imaging to detect exercise-induced microvascular adaptations in future studies.

## Introduction

Within the last few years, optical coherence tomography angiography (OCTA) has contributed insight to various retinal diseases by qualitative visualization of blood flow in the back of the eye.[1] One of the major challenges ahead is the definition of reliable quantitative OCTA measurements in health and disease since information on blood flow in the retinal vasculature is of major interest for early and refined diagnosis of not only ocular but also systemic diseases.[2–3] In this regard, there are technical issues to address such as OCT hardware, software algorithms and thresholding. Moreover, physiological intra- and extra-ocular parameters affecting the retinal vasculature are currently under (re-) investigation using highly sensitive OCTA.

Since OCTA algorithms detect blood flow based on the OCT signal scattered or reflected by moving red blood cells, one important parameter recently reported by Yang and colleagues is the hematocrit level, which significantly affects the flow signal particularly in narrow capillary vessels localized in the macula or at the optic nerve head (ONH).[4] Intraocular pressure change is an example of an intraocular parameter that influences peripapillary flow density (FD) in OCTA.[5] Other transient systemic factors, such as changes in oxygenation may also have a significant impact on FD measurements.[6] In addition, exogenous vasoactive stimulants such as caffeine have been shown to cause a reduction in macular flow area and FD.[7–9] Moreover, endogenous vasoactive factors can be anticipated to influence retinal perfusion in OCTA since a decrease of peripapillary and parafoveal blood flow immediately after intense physical activity has been reported.[10]

Recently, our group contributed data on long-term changes in ocular perfusion measured by OCTA in response to a controlled physical training intervention.[11] Thus, we hypothesized that regular physical activity and physical fitness might affect OCTA measures in healthy adults.

Therefore, the aim of this study was to evaluate if and to what extent physical fitness affects OCTA measures of ONH and macular blood perfusion in healthy adults.

## Methods

### Demographics

Sixty-five young healthy moderately trained female and male students (all Caucasian, mean age 21.52 ± 2.20 years, 43 females) of the University’s Physical Education Department were recruited at the Institute of Sports Medicine of the University Hospital Muenster in May 2017 as reported elsewhere.[11] All participating students had self-assigned to a recurrent seminar of the department in a ‘first-come first-served’ process without further selection.

All investigations were performed in accordance with the declaration of Helsinki and after the approval of the ethical committee of the medical association Westfalen-Lippe and the Westphalian Wilhelms-University of Muenster (project-no. 2013-231-f-S, study acronym SPORTIVA). Prior to subjects’ participation in the study, written informed consent was obtained. Inclusion criteria were age > 18 years, a health certificate as mandatory to study at the University’s Physical Education Department and a valid maximal performance exercise test (see below). Participants with diagnosis of any cardiovascular disease, use of cardiovascular medication or diagnosis of arterial hypertension were not eligible to participate. Any ocular pathology in the medical history as well as any pathologic alteration in structural OCT scans led to exclusion. Since axial length affects OCTA measurements of FAZ due to magnification effects particularly in higher aberrations of axial length [12], participants with spherical equivalent values higher than +3 diopters (D) and lower than -3 D were also excluded, since axial length strongly correlates with the refractive error.[13,14]

### Exercise test procedures

All participants performed a standardized incremental continuous running test (ICRT, as maximal performance test) to determine their individual fitness level. The test provides information on the individual anaerobic (lactate) threshold (IAT, given as running speed [V] at IAT) and maximal exercise capacity (given as maximal running speed, V_max_). The test was performed indoors at ambient temperature (18–22°C) on a synthetic 200 m running track in groups of 4 individuals as described elsewhere.[15–17] Subjects were fitted with heart rate (HR) monitors combined with a wireless receiver module (Acentas, Muenster, Germany). The test started at 8.0 km·h^-1^, increasing by 2.0 km·h^-1^ every 3 minutes until total exhaustion of the participant. The pace was indicated by an automated acoustic device. For blood lactate concentration measurements (Biosen S-line, EKF Diagnostics, Magdeburg, Germany), blood was sampled from participants’ earlobes after each interval (3 min). Performance at individual lactate threshold (baseline lactate + 1.5 mmol·L^-1^) was calculated using Winlactat software version 5.0.0.54 (Mesics, Muenster, Germany).[18–20] Hematocrit values (epoc handheld analyzer, Alere, Cologne, Germany) were assessed in twelve randomly selected participants.

### Imaging and image analysis

Subjects had been at rest before OCTA images were taken and were instructed to refrain from physical activity on the day of imaging. Additionally, the consumption of neither caffeine nor nicotine was allowed two hours prior to OCTA. Imaging was conducted with a commercial spectral domain OCT system (AngioVue, RTVue XR Avanti SD-OCT, Optovue, Fremont, CA, USA). Images were recorded from the right eye under standardized mesopic lighting conditions by an experienced operator. As there is ongoing debate in the recent literature whether circadian / diurnal changes affect OCTA measurements [21], OCTA imaging was performed in the afternoon between 4 pm and 6 pm. Volumetric scans of 304 × 304 A-scans (70,000 A-scans per second) were recorded using a light source at 840 nm. Two consecutive B-scans covering the central 3 x 3 mm^2^ field and the 4.5 x 4.5 mm^2^ ONH field were performed to compute inter-B-scan decorrelation with the split-spectrum amplitude-decorrelation angiography algorithm. The integrated software performs an automated segmentation, and displays the en-face OCTA and the structural en-face OCT image side by side. An eye tracking tool (DualTrac Motion Correction, Optovue) was used combined with an artifact removal function. For quality control, images showing insufficient signal strength (signal strength index [SSI] < 50) or an OCTA motion artifact score of three or four were excluded.[22] Automated segmentation was performed by the device according to defined reference planes as previously described.[23–24] The accuracy of segmentation was checked for all reference planes by two raters. Incorrect segmentation led to exclusion.

The foveal avascular zone (FAZ) was defined as the area inside the central border of the capillary network, which was outlined using the integrated semi-automated software tool. Slab thickness for FAZ analysis was defined as the range from the inner limiting membrane (ILM) to 9 μm above the outer plexiform layer. Macular flow density (FDM) was analyzed based on the above mentioned slab definition as well as separately in the superficial plexus (FDsM) and in the deep plexus (FDdM) of the macula.

For evaluation of the peripapillary blood flow, FD in the radial peripapillary layer of the peripapillary region (FDrPP) was determined. FD was defined as the percentage of the sample area occupied by vessel lumina following binary image reconstruction. The software AngioAnalytics (version 2017.1.0.151) measures flow area, non-flow area, and FD, reporting the relative density of flow as a percentage of the total evaluated area. FD is then calculated by first extracting a binary image of the vessels from the grey scale OCTA en-face image, and then computing the percentage of pixels of vessels in the defined sector based on the binary image.[25] Macular thickness was extracted for all participants using the proprietary software tool. Choriocapillaris (CC) blood flow was quantified using the CC decorrelation signal index approach. The setting for CC imaging is a ~ 20 μm-slab between 9 μm and 31 μm below the inner retinal pigment epithelium reference. CC data were exported and further evaluated using ImageJ (Version 1.51n, National Institutes of Health, USA). Images were converted into grey scale attributing each pixel to a value that represents the strength of the decorrelation signal. CC decorrelation signal index was defined as the average decorrelation value of all pixels in the en-face CC angiogram.[26] All data were extracted and analyzed blinded to participants’ characteristics.

### Statistical methods

Statistical analyses were performed using Prism 8.1 (GraphPad Software, La Jolla, USA). Data are presented as n (%), mean ± SD or median [interquartile range] in case of non-normal distribution. Data were tested for normal distribution using Kolmogorov-Smirnov test. Correlation analysis was performed using Pearson Product-Moment Correlation. For OCTA parameters showing statistically significant correlations with physical fitness measures, multiple linear regression was performed. P-value was set to 0.05.

## Results

Participants anthropometric, OCTA and exercise parameters are presented in Table 1. Six of 65 participants had to be excluded due to incomplete examination records. Participants’ submaximal and maximal performance capacity determined as running speed at the individual anaerobic (lactate) threshold (speed at IAT) and maximal running speed (V_max_, speed immediately before test termination due to fatigue) were comparable to previously reported data in similar age groups (Table 1).[17]

**Table 1:** Participants’ anthropometric, optical coherence tomography angiography (OCTA) and exercise parameters.

**Anthropometric data (n = 59)**		**Female (n = 42)**	**Male (n = 17)**
Age (years)	21.00 [20.00–23.00]	21.00 [20.00–22.00]	21.00 [21.00–24.00]
Height (cm)	174.0 ± 7.04	171.2 ± 5.58	181.0 ± 5.17
Weight (kg)	67.30 ± 9.39	63.17 ± 6.33	77.50 ± 7.82
BMI (kg·m^-2^)	22.16 ± 2.20	21.56 ± 1.91	23.65 ± 2.20
SE (D)	0.00 [-1.00–0.50]	0.00 [-1.0–0.50]	-0.71 ± 1.09
**OCTA parameters (n = 59)**			
FAZ (mm^2^)	0.24 ± 0.11	0.27 ± 0.11	0.16 [0.11–0.20]
FDM (%)	60.38 ± 1.84	60.32 ± 1.77	60.52 ± 2.05
FDsM (%)	52.26 ± 1.89	52.02 ± 1.81	52.83 ± 2.03
FDdM (%)	53.65 [51.35–54.85]	53.64 ± 2.07	51.58 ± 2.90
FDrPP (%)	50.11 ± 2.09	50.39 ± 1.96	49.42 ± 2.30
CCDI (pixel)	118.4 [117.0–121.0]	117.8 [116.1–119.8]	121.0 [117.3–121.2]
Macular thickness (μm)	333.4 ± 12.26	334.8 [323.0–339.6]	338.8 ± 12.95
**Exercise parameters (n = 59)**			
V_max_ (km·h^-1^)	15.40 [13.90–16.90]	14.55 [13.65–15.70]	17.74 ± 1.32
IAT (km·h^-1^)	10.91 ± 1.12	10.61 ± 1.04	11.64 ± 0.96

Data are presented as mean ± SD or median [interquartile range] in case of non-normal distribution. Body-mass-index (BMI). Spherical equivalent (SE). Foveal avascular zone (FAZ) area. Macular flow density (FDM). Macular flow density in the superficial plexus (FDsM) and in the deep plexus (FDdM). Flow density in the radial peripapillary capillary layer of the peripapillary region (FDrPP). Choriocapillaris (CC) decorrelation signal index (CCDI). Individual anaerobic (lactate) threshold (IAT) given as running speed [V] at IAT. Maximal exercise capacity (given as maximal running speed, V_max_).

**Fig 1:**
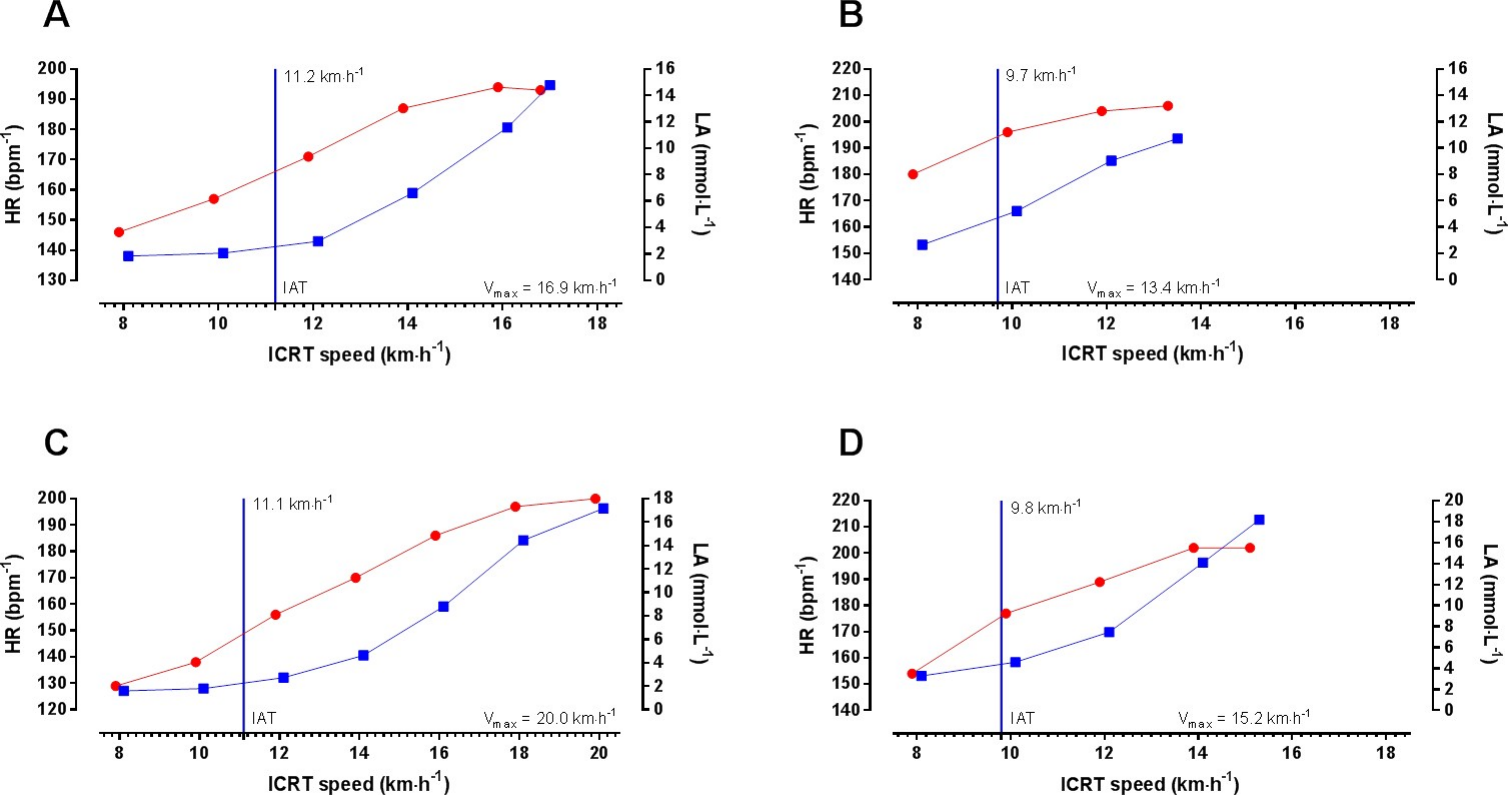
Exemplary presentation of exercise parameters. **[A]** Performance of a 20-year-old female participant. Participant’s individual anaerobic threshold (IAT) was 11.2 km·h^-1^ (upper level compared to sex-specific mean). **[B]** Performance of a 21-year-old female participant. Participant’s individual IAT was 9.7 km·h^-1^ (lower level compared to sex-specific mean). **[C]** Performance of a 21-year-old male participant. Participant’s IAT was 11.1 km·h^-1^ (upper level compared to sex-specific mean). **[D]** Performance of a 24-year-old male participant. Participant’s IAT was 9.8 km·h^-1^ (lower level compared to sex-specific mean). Exercise heart rate (HR, ■) and blood lactate concentration (LA, ●) are shown at the different Incremental Continuous Running Test (ICRT) stages given as km·h^-1^. IAT is represented as vertical blue line.

Exemplary performance data including the determination of the IAT and V_max_ of two female and two male participants is presented in Fig 1. The exhaustive nature of the maximal performance exercise test is documented by maximal heart rate of up to ~ 200 bpm^-1^ and blood lactate concentration of up to ~ 18.0 mmol·L^-1^ (Fig 1). Since performance at IAT is a parameter associated with aerobic performance and V_max_ depends on aerobic and anaerobic capacity, both parameters showed a significant but not perfect correlation (r = 0.763; p < 0.0001).

No signs of pathologic ocular alteration were observed in structural OCT scans or OCTA images of any analyzed individual and parameters obtained were comparable to published data reporting on healthy individuals of identical age.[25] SSI of all images was ≥ 50. Segmentation was correct for all reference planes in all analyzed participants with 100% agreement between both raters for all eyes analyzed.

**Fig 2:**
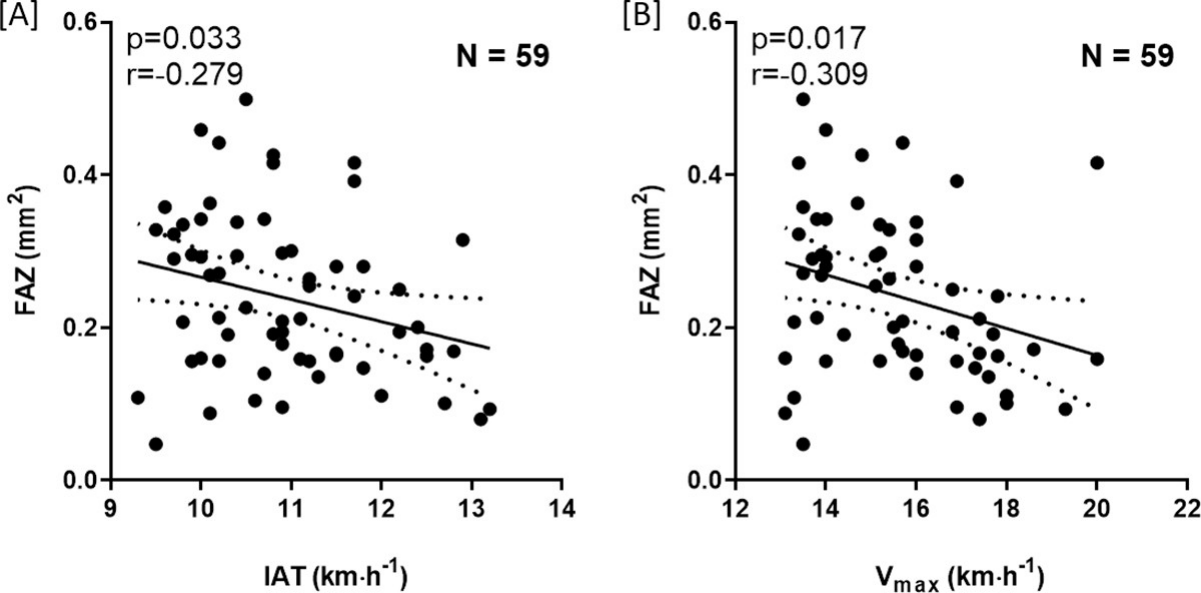
Scatter plots of individual anaerobic lactate threshold (IAT) **[A]** and maximal exercise capacity (V_max_) **[B]** and the foveal avascular zone (FAZ). Individual data points are shown with 95% CI and linear regression.

Results of the initial correlation analysis of physical performance (IAT and V_max_) with OCTA parameters are presented in Table 2. Significant inverse correlations of the FAZ area and V_max_ as well as IAT were detected (V_max_, r = -0.309, p = 0.017; IAT, r = -0.279, p = 0.033). No other OCTA parameters were significantly associated with exercise performance. Scatter plots and linear regressions showing the correlation between FAZ area and performance parameters (i.e. IAT and V_max_) are presented in Fig 2.

**Table 2:** Correlation of individual anaerobic lactate threshold (IAT) and maximal exercise capacity (V_max_) and optical coherence tomography angiography (OCTA) parameters.

Pearson‘s correlation		
	Overall (n = 59)	
Exercise parameter	r	p
**IAT (km·h**^**-1**^**)**		
FAZ (mm^2^)	-0.279	**0.033**
FDM (%)	-0.109	0.413
FDsM (%)	-0.055	0.680
FDdM (%)	-0.004	0.977
FDrPP (%)	-0.095	0.475
CCDI (pixel)	0.144	0.389
**V**_**max**_ **(km·h**^**-1**^**)**		
FAZ (mm^2^)	-0.309	**0.017**
FDM (%)	0.007	0.960
FDsM (%)	0.1057	0.426
FDdM (%)	-0.069	0.603
FDrPP (%)	-0.226	0.085
CCDI (pixel)	0.171	0.194

Significant p-values (p < 0.05) are indicated in bold. Foveal avascular zone (FAZ) area. Macular flow density (FDM). Macular flow density in the superficial plexus (FDsM) and in the deep plexus (FDdM). Flow density in the radial peripapillary capillary layer of the peripapillary region (FDrPP). Choriocapillaris (CC) decorrelation signal index (CCDI).

A multivariate regression analysis was performed to analyze the influence of IAT, V_max_, anthropometric data, SE and macular thickness on FAZ area. In the presented model, only IAT and SE contributed significantly to the regression (Table 3) (IAT: β = 0.111, p = 0.032; SE: β = 0.127, p = < 0.0001). Notably, inclusion of further OCTA parameters into the model did not affect the association of IAT with FAZ area.

**Table 3:** Multivariate regression analysis of foveal avascular zone (FAZ) area and exercise parameters, anthropomorphic data and macular parameters.

	Variable	Estimate	Standard Error	Β0	p-value
β0	Intercept	2.934	3.778	1.000	0.441
β1	IAT (km·h^-1^)	-0.033	0.015	0.111	**0.032**
β2	V_max_ (km·h^-1^)	0.023	0.013	0.058	0.089
β3	Sex (male)	-0.099	0.050	-0.094	0.053
β4	Height (cm)	-0.013	0.021	-0.991	0.544
β5	Weight (kg)	0.013	0.027	0.988	0.619
β6	BMI (kg·m^-2^)	-0.051	0.082	-0.993	0.532
β7	Age (years)	0.007	0.005	0.078	0.170
β8	SE (D)	-0.069	0.010	0.127	**< 0.0001**
β9	Macular thickness (μm)	-0.0009	0.001	-0.264	0.414

Body-mass-index (BMI). Spherical equivalent (SE). Individual anaerobic (lactate) threshold (IAT) given as running speed [V] at IAT. Maximal exercise capacity (given as maximal running speed, V_max_). Data were 1/y transformed before entering the model. Significant p-values (p < 0.05) are indicated in bold.

Of note, analysis also revealed an association between choriocapillaris decorrelation signal index (CCDI) and age, an association which has already been reported in previous histologic studies suggesting that histologically normal maculae show a decrease in density and diameter of choriocapillaris vessels with advancing age.[27]

## Discussion

The current study evaluated if OCTA measurements of ocular blood flow at the posterior pole are associated with physical performance measures in young healthy adults. We found that physical fitness defined by running speed at individual anaerobic lactate threshold (IAT) was inversely correlated with the foveal avascular zone (FAZ) area.

The more precisely and unaffected from influencing factors quantitative OCTA imaging can be performed, the more effectively and sensitively natural history studies and interventional clinical trials can detect changes in ocular perfusion that may be associated with onset or progression of disease. In this regard, the identification of intra- and extra-ocular factors, which affect blood flow properties and thus OCTA measurements is of central interest.[4–10] We have recently reported that OCTA measurements can be altered by a physical exercise intervention. After a controlled short term (4-weeks) high-intensity interval training period, FDsM and FDnhPP values were changed and a strong effect on the FAZ area was observed, which was reduced by ~ 14%.[11] The current study provides evidence that FAZ area in OCTA images of participants with higher physical fitness was smaller compared to those with lower fitness. Other OCTA parameters were not significantly associated with exercise performance. Of note, we initially detected correlations of FAZ with both determined exercise parameters, speed at IAT and maximal exercise performance (determined as V_max_). Both exercise parameters are strongly correlated and depend largely on cardiopulmonary fitness attained by regular physical activity and exercise training. While testing methods for maximal exercise performance may (at least in part) be actively or involuntarily influenced by the tested individual and the observer, blood lactate-based measures such as the IAT are considered as reliable and objective parameters for endurance exercise performance. They can neither be influenced by the participant nor by the trainer and are determined after completion of the test. It is important to note that our study group included females and males not matched for physical fitness. However, we did not detect an effect of sex on the FAZ area although, in the multivariate analysis, sex nearly missed statistical significance. In addition, subgroup analysis of females and males did not show significant correlations between IAT/ Vmax and FAZ. With regards to these results and the unbalanced sex-ratio in our cohort, future studies are required to determine the influence of sex and physical fitness on OCTA parameters. This is also of relevance since there is ongoing discussion in the current literature concerning potential sex effects on FAZ area.[12,28]

With respect to the interpretation of OCTA-derived inter-individual FAZ data, it is important to note that current commercial OCTA devices do not correct area measurements for changes in image size magnification. Thus, the actual size of the FAZ area imaged depends on the combined magnification of the camera and the analyzed eye. Garway-Heath et al. compared different methods to correct for the magnification of the eye and concluded that using axial length to correct for magnification error was a practicable approach.[29] Consistently, several recent OCTA studies suggested a strong impact of axial length on FAZ area measurement if scan size was not corrected for differences in ocular magnification.[12,28] Sampson and colleagues, reporting on a study population comparable to our group, suggested that axial lengths in the range of 23.29–24.33 mm would correspond to less than 5% variation in FAZ area measurements.[29] Lindeman and co-workers reported an average error in FAZ area determination of 8.29% from analysis of a study population with a mean axial length of 24.04 ± 1.25 mm (range: 21.45–27.45 mm).[12] Our study did not correct OCTA FAZ measurements for optical magnification since an automated correction method is not yet implemented in currently available devices. To overcome this limitation, only participants with a spherical equivalent ± 3 D were eligible for the present study and, since the spherical equivalent strongly correlates with axial length, spherical equivalent was included in the multivariate analysis of FAZ area. Notably, most participants in our study were emmetropic with a spherical equivalent of < 0.5 D and only five participants showed a mean refractive error of -0.8 D.

So far, a number of studies have reported normative data for FAZ area size and vascular densities based on OCTA imaging in healthy individuals of different age ranges. A subgroup analysis of macular OCTA data of subjects between 20–39 years of age reported data very similar to our findings.[25] Multivariate regression in our study showed no effect of age on FAZ area, most likely because the age range of our study was quite narrow. However, study results on the correlation of age and OCTA parameters such as FAZ have been conflicting, which might also be based on the existence of so far unconsidered parameters such as physical fitness.[12,30]

Since OCTA data with information on regular physical activity or training status of the analysed participants is missing from the literature, we can only interpret our data within the findings of our own analysis. Interestingly, both performance parameters, IAT and V_max,_ correlated with FAZ area size in the univariate linear regression model. However, only IAT reached statistical significance in the multivariate regression analysis. This observation might indicate that aerobic exercise capacity or regular exercise training performed to improve aerobic performance affects FAZ measurements to a greater extend then maximal exercise performance capacity. Compared to other known effectors, the effect of individual fitness on the FAZ area in OCTA appears rather moderate, particularly in comparison to the known effect of the spherical equivalent. However, also moderate effects may have biological relevance and this finding should also be interpreted with respect to the narrow range of physical fitness in our young and healthy population. The range of physical fitness in the overall population (ranging from sedentary and older individuals to young highly-trained individuals) is considerably large and future studies are needed to investigate the effect of physical fitness and regular physical activity on OCTA measurements including the FAZ area in population-based studies. Of note, no associations were detected for the investigated FD parameters, indicating that FAZ parameters are sensitive to physical activity and training over the entire range of physical performance capacity while significant effects on FD may only be detected in response to physical training in sedentary individuals. Notably, long-term training effects seem to differ from acute physical activity effects in that FDrPP, which has been shown to decrease immediately after exercise, was not associated with physical fitness in the current analysis.[10,11]

The above described observations may be explained by different effectors including local factors causing vasodilation. For instance, retinal vasomotor function is, as part of the cerebral circulation, largely dependent on local nitric oxide (NO) production.[31,32] Asymmetric dimethylarginine (ADMA), an endogenous NO synthase inhibitor, is a known independent cardiovascular risk factor and has been shown to influence arteriolar and venule diameter.[33] A comparison of obese and moderately-trained subjects as well as athletes showed a trend to lower ADMA in individuals with higher physical fitness.[34] This observation may point towards an NO-mediated mechanism for fitness-dependent blood flow measurements in the FAZ area.

Several limitations may exist for the current study. A significant effect of haematocrit on the decorrelation signal using 45%, 40% and 32% haematocrit blood samples has been described in an ex-vivo model.[4] In our study, haematocrit values were determined in twelve randomly selected participants without significant differences in haematocrit values. However, this sample may not allow to fully exclude any effects of the haematocrit in our study. The process of thresholding in OCT technology (each pixel in the OCTA en-face image is attributed a value of 0 if the pixel value in the OCT B-scan is below the threshold level), which aims at suppressing artefacts caused by noise, may have affected the current results.[35]. Furthermore, axial length measurements were not performed. However, with regard to the spherical equivalent values in our study sample, it seems not conceivable that axial length affected our measurements beyond the range of five percent as mentioned above.[28] The current study involved a group of moderately-trained healthy young individuals in a narrow age range. Compared with the age-matched general population, our participants mean performance was slightly elevated and comprised more females than males. Therefore, no direct conclusions should be drawn for patients with ocular pathologies or subjects of other age groups or subjects with inferior physical activity levels. Blood pressure and heart rate during OCTA measurements were not registered. Besides, the appearance of OCTA image data strongly depends on details of the OCT device, scan protocols, signal processing, and algorithms used to generate OCTA information from structural OCT data.[36] Therefore, the results presented must not be directly transferred to other OCTA devices.

In conclusion, we provide evidence that OCTA measurements of FAZ are associated with physical fitness. In detail, smaller FAZ areas were observed in individuals with higher aerobic exercise capacity determined by IAT.

At this point, future OCTA studies are not required to be corrected for physical activity patterns. The detected effect was moderate and based on a young and healthy group of moderately trained individuals. Follow-up studies must confirm the presented results and aim to include larger groups with wider age ranges and greater diversity in physical fitness.

The better we understand which intra- and extra-ocular variables affect OCTA measurements, the more reliable and more exact findings will be provided, also with regard to the association of retinal perfusion and pathology and the determination of potential surrogate markers at early asymptomatic disease stages.
